# The health system and health impacts of primary healthcare reform in China: A systematic review

**DOI:** 10.1093/eurpub/ckac129.538

**Published:** 2022-10-25

**Authors:** C Cai, S Xiong, C Millett, M Tian, T Hone

**Affiliations:** 1 Public Health Policy Evaluation Unit, Imperial College London, London, UK; 2 The George Institute for Global Health, University of New South Wales, Sydney, Australia; 3 Global Health Research Centre, Duke Kunshan University, Kunshan, China; 4 School of Public Health, Harbin Medical University, Harbin, China; 5 National School of Public Health, NOVA University, Lisbon, Portugal

## Abstract

**Background:**

China has undergone a comprehensive primary healthcare(PHC) reform since 2009 aiming to deliver accessible, higher-quality, and equitable healthcare. However, there is limited understanding of the effectiveness of this reform. This systematic review synthesizes evidence on health system and health impacts of this reform.

**Methods:**

We searched 13 international databases and three Chinese databases for quantitative studies assessing the impacts of this reform published between January 2009 and March 2020. We searched for studies in English or Mandarin. Eligible study designs were RCTs, quasi-experimental studies and controlled before-after studies. We included studies that: assessed PHC policies since 2009; had geographical, temporal or population comparators; and assessed any outcome measures of health expenditures, health service utilisation, quality of care or health outcomes. Study quality was assessed using ROBINS-I, and results synthesized narratively. PROSPERO: CRD42021239991.

**Results:**

Of 35,480 titles, 37 studies were included (27 in English and ten in Mandarin). Eight were considered at low risk of bias. The 37 studies covered all major PHC policies since 2009, but mostly focused on the essential medicine (N = 15) and financing (N = 10). The quantity and quality of studies on service delivery policies(e.g., family physician and essential health services), were low(N = 3,with moderate or serious risk of bias). 17 studies found that the PHC reforms promoted primary care utilisation. Its impacts on quality and health improvement appear limited to people with chronic diseases(N = 11). Evidence on primary care costs and OOPs were not clear. Some evidence showed that the reforms were pro-equity with benefits accrued in disadvantaged regions and groups.

**Conclusions:**

Comprehensive PHC reforms can deliver some benefits related to utilisation and health for high-risk and vulnerable populations. Policymakers should continue to prioritize PHC to achieve Universal Health Coverage.

**Key messages:**

• The finding suggests that large-scale and comprehensive primary healthcare reforms can deliver benefits related to utilisation and health for high-risk and vulnerable populations.

• Future research should include more robust study designs and seek to better understand the impact of major PHC reforms on quality of care, health outcomes and equity.

